# Anti-rotaviral effects of *Glycyrrhiza uralensis* extract in piglets with rotavirus diarrhea

**DOI:** 10.1186/1743-422X-9-310

**Published:** 2012-12-18

**Authors:** Mia Madel Alfajaro, Hyun-Jeong Kim, Jun-Gyu Park, Eun-Hye Ryu, Ji-Yun Kim, Young-Ju Jeong, Deok-Song Kim, Myra Hosmillo, Kyu-Yeol Son, Ju-Hwan Lee, Hyung-Jun Kwon, Young Bae Ryu, Su-Jin Park, Sang-Ik Park, Woo Song Lee, Kyoung-Oh Cho

**Affiliations:** 1 Biotherapy Human Resources Center, College of Veterinary Medicine, Chonnam National University, Gwangju, 500-757, Republic of Korea; 2 Chonnam National University Veterinary Teaching Hospital, Gwangju, 500-757, Republic of Korea; 3 Eco- Friendly Biomaterial Research Center and AI Control Material Research Center, Korea Research Institute of Bioscience and Biotechnology, Jeongup, 580- 185, Republic of Korea; 4 Present address: Department of Clinical Pathology, College of Medicine, Seonam University, Namwon, 590-711, Republic of Korea

**Keywords:** Rotavirus, Enteritis, *Glycyrrhiza uralensis* extract, Anti-rotaviral drug

## Abstract

**Background:**

Since rotavirus is one of the leading pathogens that cause severe gastroenteritis and represents a serious threat to human and animal health, researchers have been searching for cheap, safe, and effective anti-rotaviral drugs. There is a widespread of interest in using natural products as antiviral agents, and among them, licorice derived from *Glycyrrhiza* spp. has exerted antiviral properties against several viruses. In this study, anti-rotaviral efficacy of *Glycyrrhiza uralensis* extract (GUE) as an effective and cheaper remedy without side-effects was evaluated in colostrums-deprived piglets after induction of rotavirus diarrhea.

**Methods:**

Colostrums-deprived piglets were inoculated with porcine rotavirus K85 (G5P[7]) strain. On the onset of diarrhea, piglets were treated with different concentration of GUE. To evaluate the antiviral efficacy of GUE, fecal consistency score, fecal virus shedding and histological changes of the small intestine, mRNA expression levels of inflammation-related cytokines (IL8, IL10, IFN-β, IFN-γ and TNF-α), signaling molecules (p38 and JNK), and transcription factor (NFκB) in the small intestine and spleen were determined.

**Results:**

Among the dosages (100-400 mg/ml) administrated to animals, 400 mg/ml of GUE cured diarrhea, and markedly improved small intestinal lesion score and fecal virus shedding. mRNA expression levels of inflammation-related cytokines (IL8, IL10, IFN-β, IFN-γ and TNF-α), signaling molecules (p38 and JNK), and transcription factor (NFκB) in the small intestine and spleen were markedly increased in animals with RVA-induced diarrhea, but dose- dependently decreased in GUE treated animals after RVA-induced diarrhea.

**Conclusions:**

GUE cures rotaviral enteritis by coordinating antiviral and anti-inflammatory effects. Therapy of this herbal medicine can be a viable medication for curing rotaviral enteritis in animals and humans.

## Background

Group A rotaviruses (RVAs) are the leading cause of gastroenteritis, malnutrition, and diarrhea in young children and animals [1]. In humans, RVAs are estimated to cause 453,000 deaths per year in children below 5 years of age, mostly in developing countries [2]. Deaths from RVA infection are most prevalent in developing nations, where patients may not always receive adequate medical attention quickly enough [3].

Currently, there are two commercially available vaccines in the market. RotaTeq (Merck) is a pentavalent human-bovine reassortant live attenuated oral vaccine, while Rotarix (GlaxoSmithKline) is a live-attenuated human RVA vaccine [4]. These vaccines appear to be promising in preventing RVA diarrhea. However, each is only effective against a particular strain of the virus, the high cost of production, and has a high probability of manifesting side effects in particular with vaccine-derived transmission of RVAs in immunocompromised patients [5].

Synthetic compounds, such as ribavirin, 3-deazaguanine, cimetidine, famotidine, dipyridamole, nifedipine, and isoprinosine, have been shown to inhibit RVA infection [6-8]. Compared with these synthetic compounds, natural compounds, such as *Diotahedral smecta*, tea and pine seed shell extract, cacao pigment, *Sophora flavescens* extract, and *Stevia rebaudiana* have been identified as ideal candidates for antirotaviral drugs in developing countries because they are effective and cheaper with minimal or without toxicity and side-effects [9-11]. Also noted were compounds found in human milk and soy infant formulation [12-14] that were effective in inhibiting rotavirus infection. Potential components found in high levels in soy-based infant formulation were isoflavones [14]. *In vitro* study revealed that genistin and mixtures of isoflavones can inhibit rotavirus infection by modulating virus attachment and post-binding step [14]. High levels of antiviral activity were found to be related with mucin found in the human milk [12]. Both *in vitro* and *in vivo* studies showed that variation in milk mucin glycoproteins may be correlated with different levels of protection against infection with gastrointestinal pathogens [12]. Lactadherin, a mucin-associated glycoprotein was also found to specifically inhibit rotavirus replication both *in vitro* and *in vivo*[13].

Licorice, dried and processed *Glycyrrhiza radix*, has been used for the treatment of peptic ulcer disease, constipation, cough, and other diseases since antiquity [15,16]. In addition, licorice extracts have generally been recognized as safe and are used as flavoring and sweetening agents for tobaccos, chewing gums, candies, toothpaste, and beverages [16]. The bioactive constitute of licorice root are saponins, flavonoids, isoflavones, coumarins, stilbenoids, and some miscellaneous compounds [15]. These compounds exert many useful pharmacological properties that include anti-cancer, anti-diabetic, anti-inflammatory, anti-malaria, anti-bacterial, antioxidant, and estrogenic activities [15,17]. The compounds extracted from *Glycyrrhiza* species showed antiviral activities via inhibiting virus absorption and replication such as influenza virus, severe acute respiratory syndrome (SARS) coronavirus, hepatitis A-C viruses (HAV-HCV), Epstein Barr virus, human immunodeficiency virus (HIV), and Japanese encephalitis virus [16,18]. Moreover, these compounds are known to modulate virus-induced inflammatory response [17,19]. In our previous study [20], polyphenol compounds from the roots of *Glycyrrhiza uralensis* (*G. uralensis*) exerted *in vitro* anti-RVA activity by inhibiting both viral absorption and viral replication. Another *in vitro* study also suggests that 18β-glycyrrhetinic acid, the aglycone product of glycyrrhizin hydrolysis can inhibit rotavirus entry into the cells [21]. However, there is little evidence whether this compound inhibit RVA infection in the animal models*.*

The aim of this study is to investigate the influence of the *G. uralensis* extract (GUE) on RVA-induced diarrhea, fecal RVA shedding, RVA-induced histological lesion changes, RVA-induced cytokine expression, and RVA-induced cellular signaling events in colostrums-deprived piglets. The results of this study suggest that the GUE cures rotaviral enteritis by coordinating antiviral and anti-inflammatory effects.

## Results

### Influence of GUE on RVA-induced diarrhea

**Figure 1:**
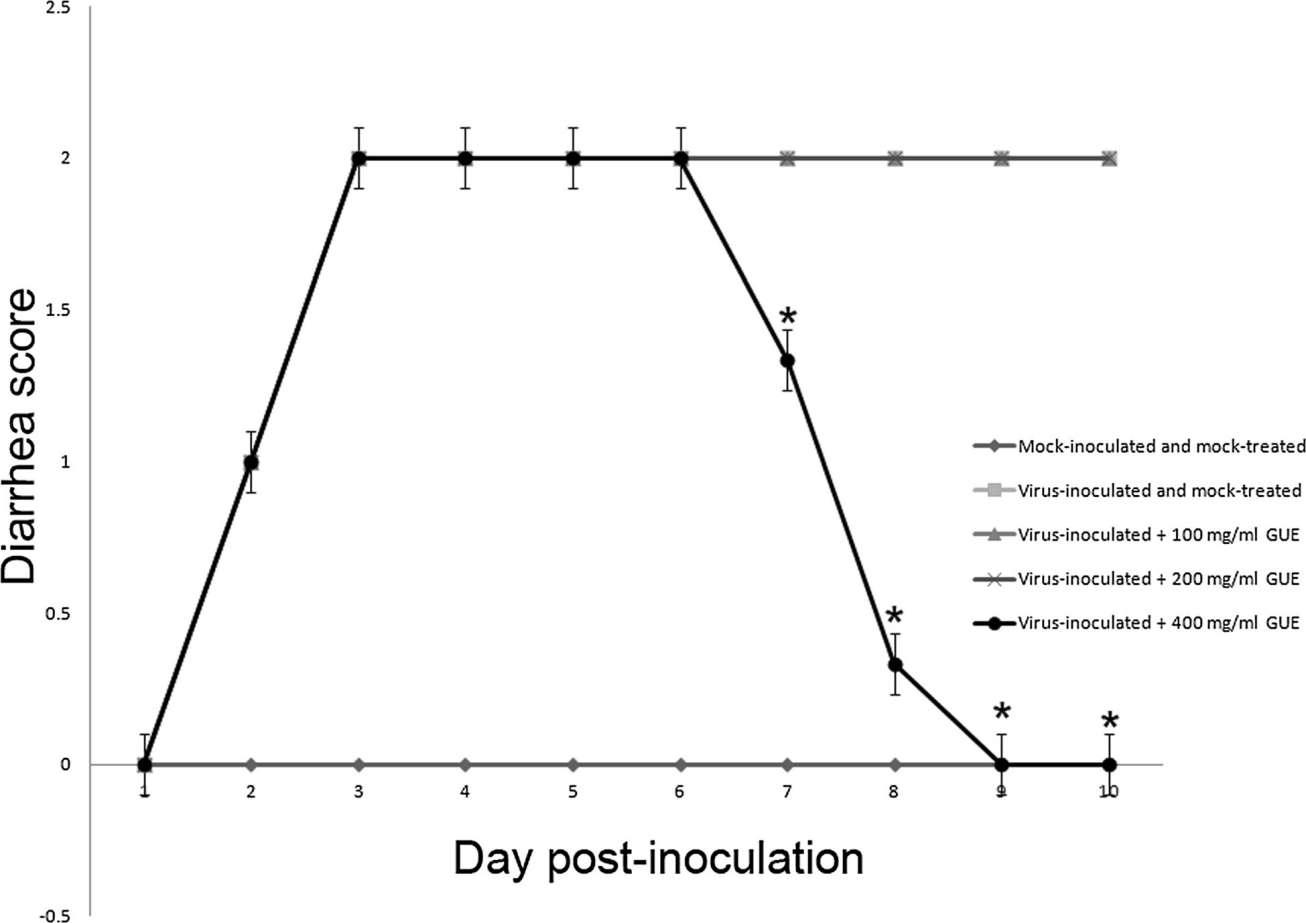
**Duration and fecal consistency score of colostrums-deprived piglets treated with the *Glycyrrhiza uralensis *extract (GUE) after induction of rotaviral diarrhea.** Values are mean ± S.D. (*n*=3) (*p<0.05).

To determine the effective dose of GUE for RVA-induced diarrhea, colostrums-deprived piglets were orally given with 100 mg/ml, 200 mg/ml, or 400 mg/ml. Control piglets with mock-inoculation and mock-treatment did not show diarrhea throughout the experimental period, whereas piglets inoculated with RVA alone showed diarrhea at day post-inoculation (DPI) 2 and diarrhea persisted to the termination of the experiment. All piglets treated with 100 mg/ml and 200 mg/ml continuously showed diarrhea until the termination of the experiment (Figure 1). The piglets treated with 400 mg/ml GUE exhibited decreased fecal consistency score at DPI 7, and diarrhea was cured at DPI 8 or 9 (Figure 1).

### Influence of GUE on fecal RVA shedding

**Figure 2:**
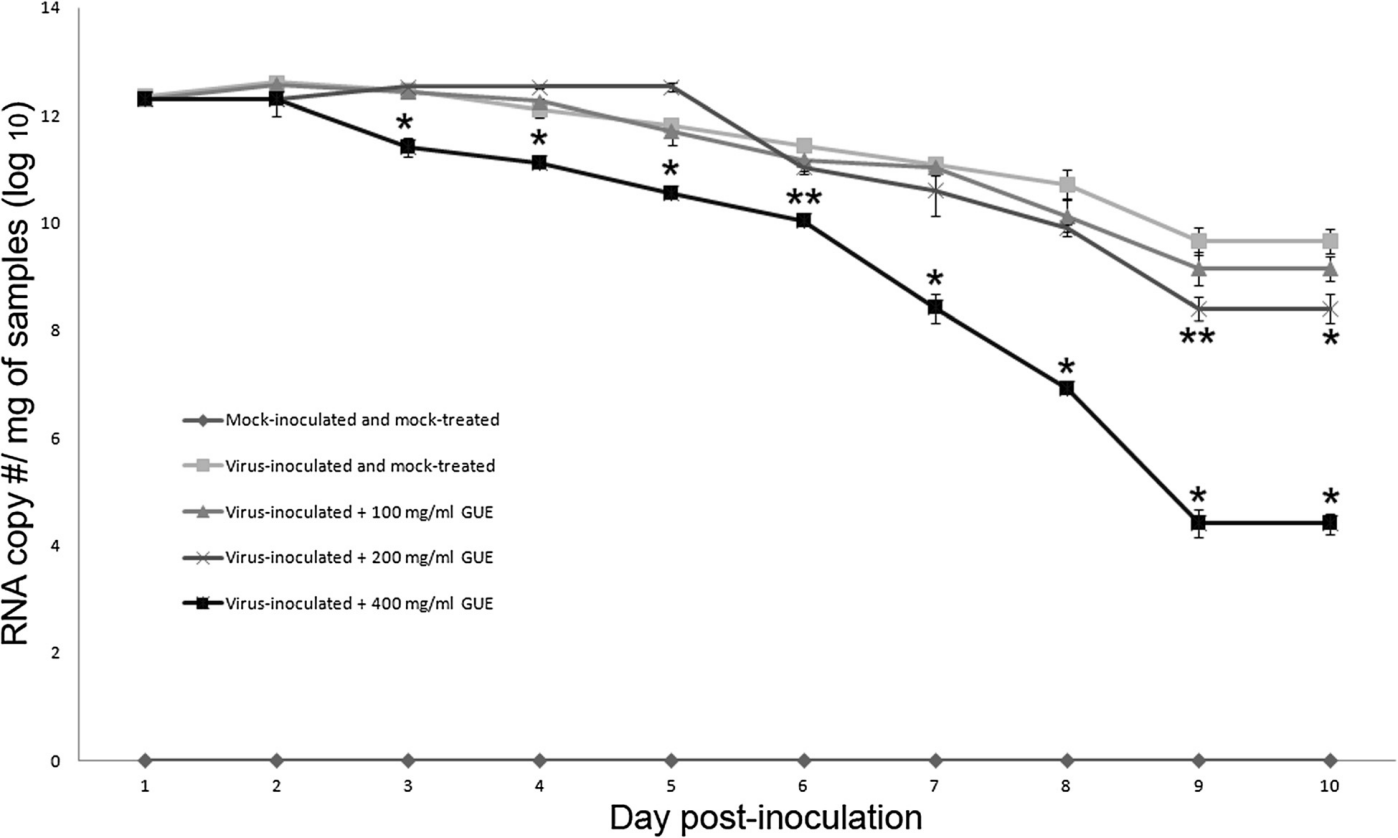
**Quantification of viral RNA copy numbers by SYBR Green real-time RT-PCR in the fecal samples of mock-treated and *Glycyrrhiza uralensis *extract (GUE).** Values are mean ± S.D. (*n*=3). The asterisk indicate significant differences (*p<0.05; **p<0.01) compared to the positive control.

To evaluate the influence of GUE on the fecal RVA shedding, the viral RNA copy numbers in the fecal samples were individually quantified, enabling the comparison between the mocked-treated and treated groups. Piglets treated with 100 mg/ml and 200 mg/ml GUE did not show any improvement of fecal virus shedding, which remained high until the termination of the experiment in comparison with the virus inoculated and mocked-treated piglets (Figure 2). Piglets treated with 400 mg/ml revealed a rapid decrease of viral copy numbers in the fecal samples, compared with virus inoculated and mocked-treated group (Figure 2).

### Influence of GUE on RVA-induced histological lesion changes in the small intestine

**Figure 3:**
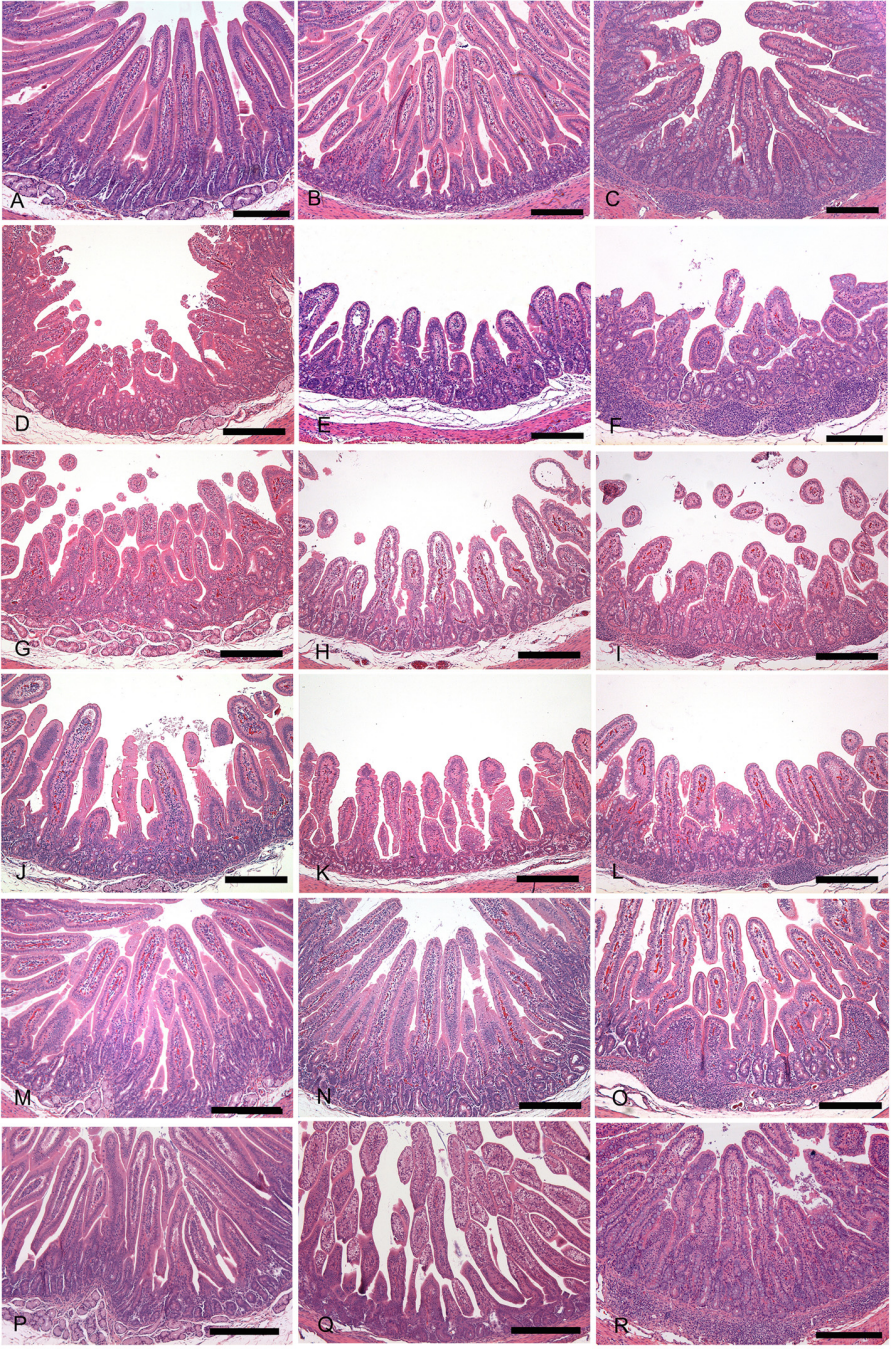
**Histopathological changes of small intestine sampled from control and G*lycyrrhiza uralensis *extract (GUE) treated groups.** (**A**-**C**) Control piglet shows unaltered duodenum (**A**), jejunum (**B**) and ileum (**C**) with long and slender villi. (**D**-**F**) Piglet inoculated with RVA displays severe villi atropy and crypt hyperplasia in the duodenum (**D**), jejunum (**E**) and ileum (**F**). (**G**-**I**) Piglet treated with 100 mg/ml GUE exhibits slight improvement of lesion changes in the duodenum (**G**), jejunum (**H**) and ileum (**I**). (**J**-**L**) Piglet treated with 200 mg/ml GUE reveals moderate improvement of lesion changes in the duodenum (**J**), jejunum (**K**) and ileum (**L**). (**M**-**O**) Piglet treated with 400 mg/ml GUE shows markedly restored villi and crypt in the duodenum (**M**), jejunum (**N**) and ileum (**O**). (**P**-**R**) Piglet treated with 400 mg/ ml GUE shows unaltered duodenum (**P**), jejunum (**Q**) and ileum (**R**) with long and slender villi. Hematoxyline and eosin stain. Bars = 200 μm.

To determine the influence of GUE on RVA-induced histological lesion changes in the small intestine, intestinal samples were collected from all experimental piglets. The histological changes in the small intestines sampled from each experimental group are summarized in Table 1. The mock-inoculated group showed long and slender villi with short crypts of the small intestine (Figure 3A-C), while virus-inoculated piglets had marked villi atrophy and crypt hyperplasia (Figure 3D-F). Compared to severe histological changes of small intestine from piglets inoculated with RVA alone, piglets given 100 mg/ml or 200 mg/ml GUE exhibited gradual improvement of lesion changes (Figure 3G-L). Remarkably, piglets receiving 400 mg/ml of GUE showed marked improvement of the small intestinal lesion (Figure 3M-O). Like the control group, piglets treated with various concentrations of GUE alone had normal villi and crypts in the small intestine (Figure 3P-R).

**Table 1:** Summary of the histopathological lesion changes in the small intestines sampled from each experimental group

**Experimental groups**	**Lesion score**^**a, b**^		
	**Duodenum**	**Jejunum**	**Ileum**
Mock-inoculated & mock-treated	0 ± 0.00	0 ± 0.00	0 ± 0.00
400 mg GUE treated	0 ± 0.00	0 ± 0.00	0 ± 0.00
Virus-inoculated + mock-treated	3.82 ± 0.06	3.09 ± 0.05	2.95 ± 0.10
Virus-inoculated + 100 mg GUE treated	3.18 ± 0.03***	2.86 ± 0.30	2.80 ± 0.06
Virus-inoculated + 200 mg GUE treated	2.91 ± 0.01***	2.83 ± 0.01	2.77 ± 0.03*
Virus-inoculated + 400 mg GUE treated	1.58 ± 0.08***	1.54 ± 0.02***	1.50 ± 0.05***

^a^ The intestinal changes are scored according to the average villi/crypt (V/C) ratio plus the grade of epithelial cell desquamation, which is measured as follows: V/C ratio, 0 = normal; (V/C ≥6:1), 1 = mild; (V/C = 5.0 – 5.9:1), 2 = moderate; (V/C = 4.0 – 4.9:1), 3 = marked; (V/C = 3.0 – 3.9:1), 4 = severe; (V/C ≤3.0:1), and desquamation grade, 0 = normal (no desquamation), 1 = mild (a few desquamated cells of tip villous epithelium), 2 = moderate (desquamation of upper villous epithelium), 3 = marked (desquamation of lower villous epithelium), 4 = severe (desquamation of crypt epithelium).

^b^ Values are mean ± S.D. (*n*=3). The asterisk indicate significant differences (*p<0.05; *** *p*<0.001) compared to the positive control.

### Influence of GUE on activation of p38, JNK, and NFκB

**Figure 4:**
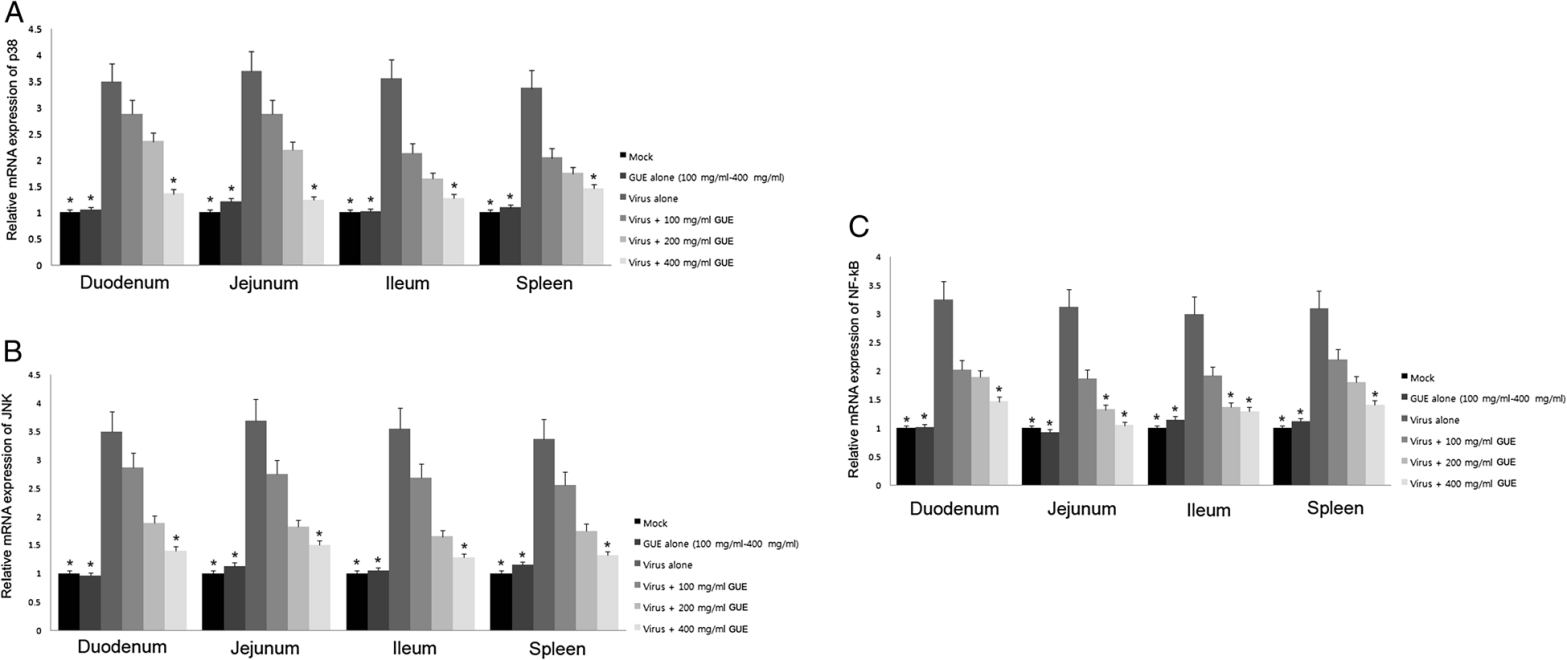
**Influence of *Glycyrrhiza uralensis *extract (GUE) on rotavirus-induced expression of p38, JNK, and NF-κB in colostrums-deprived piglets.** Comparison of mRNA expression levels of p38, JNK, and NF-κB with those of β-actin in each experimental group was determined by SYBR Green Real-time RT-PCR. Values are means ± S.D. (*n*=3) (*p<0.05).

Relative mRNA expression using specific primer pairs to p38, JNK, or NF-κB was quantified by real-time PCR assay. Samples of the duodenum, jejunum, ileum and splenocytes were collected from each group. mRNA expressions levels of p38, JNK, and NF-κB were compared with that of β-actin**.** The mock-treated piglets after RVA diarrhea had higher mRNA expression levels of p38, JNK, and NF-κB in the duodenum, jejunum, ileum, and splenocytes, compared to the normal piglets (Figure 4A-C). Dose-dependently, GUE treatment reduced these expression levels in the duodenum, jejunum, ileum, and splenocytes, and significant down-regulation was observed in the 400 mg/ml GUE treatment group (Figure 4A-C). A highly significant linear effect of GUE in reducing the mRNA expression of p38, JNK, and NF-kB was observed in the duodenum, jejunum, ileum, and splenocytes using a polynomial orthogonal contrast (data not shown). Like the control group, the piglets treated with GUE alone, regardless of its concentration (100-400 mg/ml), showed the basal levels of p38, JNK, and NF-κB expression in the duodenum, jejunum, ileum and splenocytes (Figure 4A-C).

### Influence of GUE on mRNA expression levels of IL8, IL10, IFN-β, IFN-γ and TNF-α

**Figure 5:**
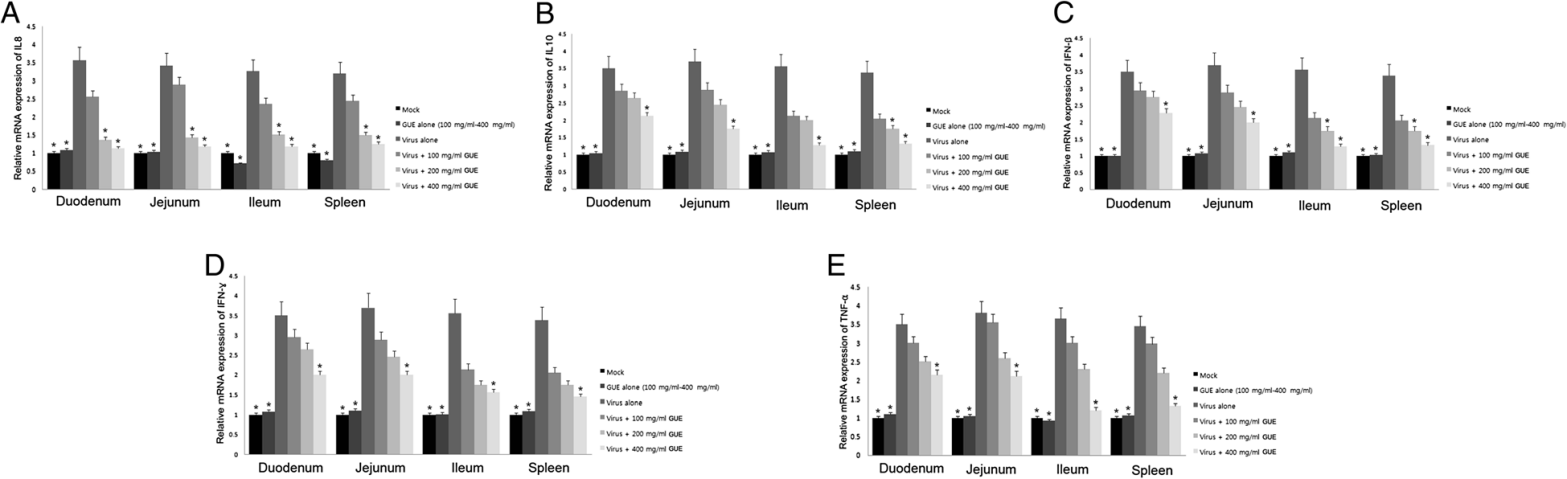
**Influence of *Glycyrrhiza uralensis *extract (GUE) on rotavirus-induced expression of IL8, IL10, IFN-β, IFN-γ, and TNF-α in colostrums-deprived piglets.** Comparison of mRNA expression levels of IL8, IL10, IFN-β, IFN-γ, and TNF-α with those of β-actin in each experimental group was determined by SYBR Green Real-time RT-PCR. Values are means ± S.D. (*n*=3) (*p<0.05).

Real-time RT-PCR assays with primer pairs specific to IL8, IL10, IFN-β, IFN-γ or TNF-α were performed with duodenum, jejunum, ileum and splenocytes sampled from each group. The relative mRNA expression levels were compared with that of β-actin. The normal or mock-inoculated and GUE-treated groups (100-400 mg/ml) showed the basal levels of IL8, IL10, IFN-β, IFN-γ, and TNF-α mRNA expression in the duodenum, jejunum, ileum, and splenocytes (Figure 5A-E). However, the mock-treated piglets after RVA diarrhea had higher mRNA levels of IL8, IL10, IFN-β, IFN-γ, and TNF-α, compared to the normal piglets. Interestingly, GUE-treated groups after RVA diarrhea showed a gradual decrease of these cytokines in a dose-dependent manner in these tissues (Figure 5A-E). Further analysis of the orthogonal contrasts showed linearity of data for reducing the mRNA expression of IL8, IL10, IFN- β, IFN- γ, and TNF- α in conjunction with the increasing amount of GUE in duodenum, jejunum, ileum, and splenocytes (data not shown).

## Discussion

In spite of the intensive efforts to prevent and control RVA infections, it is still a major global problem with high morbidity and mortality particularly in developing countries [9]. Therefore, inexpensive and effective natural compounds from food and herbal products are ideal candidates for use as preventative and therapeutic drugs against RVA diarrhea in developing countries. Previously, we demonstrated that polyphenol compounds isolated from the roots of *G. uralensis* have *in vitro* anti-RVA activity [20]. Recently, 18beta-glycyrrhetinic acid is known to inhibit rotavirus replication in culture [21]. These results prompted us to presently evaluate anti-RVA activity of GUE in an animal model.

A number of studies have been conducted using neonatal pig model in studying vaccines, passive antibody (Ab) treatments and development of immune response to rotavirus infection [22]. Moreover, the similarity in the gastrointestinal physiology and immune system of piglets and human infants [22] makes it a suitable animal model for testing the efficacy of antirotaviral drug candidates. In the present study, colostrums-deprived piglets obtained from sows by hysterectomy and maintained in isolator units were used as a RVA animal model, and this model exactly showed diarrhea at DPI 2 after oral inoculation of a porcine RVA K85 strain. We selected the porcine G5P[7] K85 strain for this study since the genotype constellation of the porcine and human rotaviruses was very similar, suggesting sharing the same ancestor [23]. Among virus-inoculated and GUE treated groups, GUE at a dose of 400 mg/ml administered to the test animals completely cured RVA diarrhea. The anti-RVA effects of GUE were also demonstrated by a significant improvement of the intestinal lesion score and of the fecal virus shedding. These results suggest that this herbal medicine may be a potent medication for curing RVA diarrhea in humans and animals. In recent studies, rotaviruses had been detected in extraintestinal organs and in serum samples of rotavirus-infected animals and humans [24]. In the future study, therefore, the effect of GUE on these points should be addressed.

Although the precise mechanisms of how the GUE cured RVA diarrhea in the colostrums-deprived piglets are unknown, diverse pathways may be participated. Of particular interest is that polyphenol compounds isolated from the roots of *G. uralensis* had inhibitory effects of RVA activities in cultured cells through blocking viral adsorption to cells and virus replication in the cells [20]. In the present *in vivo* study, GUE completely cured diarrhea and significantly improved fecal virus shedding and histological lesion changes in the small intestine. Like our *in vitro* data [20], polyphenol compounds in the GUE may inhibit virus replication in the villous epithelium of small intestine, where RVAs mainly replicate. This inhibitory activity may result in marked reduction of virus excretions from the infected cells into small intestinal lumen. Another interesting finding showed that 18β-glycyrrhetinic acid, a metabolite of glycyrrhizin reduced the yields of infectious virus and viral protein levels by interfering with rotavirus replication at a step subsequent to virus entry [21]. Another possible *in vivo* inhibitory mechanism is that polyphenol compounds in the GUE may bind the sialic acid receptor of RVA on the cell surface of the small intestinal villi [20]. Consequently, viruses released from the infected cells could not infect new epithelial cells of the small intestinal villi. Taken together, the data indicate that all these compounds in the GUE cure diarrhea by blocking RVA attachment (polyphenol compounds), entry (18β-glycyrrhetinic acid) and replication (polyphenol compounds) in the villous epithelium.

RVA infection elevates proinflammatory cytokines, such as IL1, IL8, and TNF- α, and later the Th1 cytokines, such as IL2 and IFN-β, and the Th2 cytokines IL4, and IL10 [25,26]. Activation of NFκB, p38, and JNK has been associated with virus replication and virus-induced pro-inflammatory gene expression [27,28]. However, some rotavirus strains are known to down-regulate NFκB activity through the functions of nonstructural protein NSP1 [29]. Inflammatory responses, which are the most important host defense mechanism against pathogens, can be detrimental and are often more harmful to the hosts than the inciting pathogens. In the present experiment, RVA infection increased mRNA expression levels of IL-8, IL-10, IFN-β, IFN-γ and TNF- α in the small intestine and spleen in piglets inoculated with RVA alone, whereas GUE treatment down-regulated the mRNA expression levels of these genes in the piglets after the induction of RVA diarrhea as indicated by polynomial orthogonal contrast. Moreover, NFκB, p38, and JNK genes were inhibited in the GUE-treated piglets after RVA diarrhea, but were activated in the RVA-infected piglets without treatment. These data suggested that GUE treatment modulates proinflammatory reaction, leading alleviating intestinal lesions being exacerbated by host inflammation.

It is known that glycyrrhizin down-regulates proinflammatory cytokines, and inhibits the activation of NFκB, p38, and JNK genes in virus-infected cells [27,30,31]. Notable, however, were reports that showed 18beta-glycyrrhetinic acid can induce NFκB activation and generate IL-8 secretion in RVA-infected MA 104 cells [21,32]. In the present study, proinflammatory cytokines, and its related transcription factor and signaling molecules are inhibited in the GUE-treated piglets after RVA diarrhea, but not in the RVA-inoculated piglets without treatment. Although 18beta-glycyrrhetinic acid can induce NFκB activation and generate IL-8 secretion *in vitro* and possibly *in vivo*, *in vivo* inhibitory effects of these proinflammatory reactions might be predominant by glycyrrhizin and other compounds in the GUE as well as viral nonstructural protein NSP1. The future studies need to address which compound exerts inflammatory or anti-inflammatory effects in animal models. We are currently investigating the roles of several compounds in the GUE in modulating the rotavirus infection *in vivo*.

In addition, the activation of NFκB, p38, and JNK genes is constituents of redox-sensitive signaling pathways [33,34]. Therefore, anti-oxidants had been already found to interfere with virus-induced pro-inflammatory gene expression [27]. Since glycyrrhizin is known to exert antioxidative effects [15], GUE may interfere with RVA-induced reactive oxygen species formation and then protect intestinal epithelial cells from reactive oxygen species elicited by RVA infection.

In conclusion, we show in this report that GUE cure RVA-induced diarrhea in colostrums-deprived piglets. The anti-inflammatory and antiviral effects of GUE can be attributed to the interaction of the different pharmacological active components of GUE. Since there are no currently available anti-RVA drugs for human or animal use, this anti-rotaviral drug candidate may complement the arsenal of potential drugs for the treatment of RVA enteritis, not only in animals but also in humans.

## Materials and methods

### Cell and virus

Porcine K85 (G5[P7]) strain which was originally isolated from fecal sample of a diarrheic piglet [35] was cultured in fetal rhesus monkey TF-104 kidney cell (a cloned derivative of MA-104 monkey kidney cells). Virus titer was determined by cell culture immunofluorescence assay with monoclonal antibody against the VP6 protein of the porcine rotavirus strain OSU, and was expressed as fluorescence focus units per milliliter (FFU/ml).

### Preparation of GUE

Dried roots of *G. uralensis* were extracted with methanol at room temperature as described previously [20]. It was prepared by dissolving the material in absolute ethanol to create a 1000 mg/ml stock. From the stock solution, 100 mg/ml, 200 mg/ml, and 400 mg/ml were made by diluting the stock with autoclaved distilled water.

### Animals and experimental design

Colostrums-deprived piglets were obtained from sows by hysterectomy. They were maintained in isolator units as previously described using separate isolator units for each treatment group [36]. Piglets were fed every 6 hours with a diet consisting of sterilized commercialized milk. All piglets were negative for rotavirus antibodies prior to RVA exposure. They were randomly divided into eight groups: 1) virus-inoculated and mock-treated, 2) virus-inoculated and 100 mg/ml of GUE treated, 3) virus-inoculated and 200 mg/ml of GUE treated, 4) virus-inoculated and 400 mg/ml of GUE treated, 5) mock-inoculated and mock-treated, 6) mock-inoculated and 100 mg/ml of GUE treated, 7) mock- inoculated and 200 mg/ml of GUE treated, and 8) mock-inoculated and 400 mg/ml of GUE treated. Each group contained three piglets. At 3 days of age, piglets of groups 1-4 were orally inoculated with 3 ml of the K85 strain containing a virus titer of 5 x 10^5^ FFU/ml. After DPI 2, piglets showed signs of diarrhea. Among these groups, three groups were treated four times daily with 100 mg/ml, 200 mg/ml, or 400 mg/ml of GUE for 7 days, respectively. The remaining virus-inoculated group was mock-treated. At 5 days of age, three mock-inoculated groups were treated four times daily with 100 mg/ml, 200 mg/ml, or 400 mg/ml GUE for 7 days, respectively. As the control group, piglets were mock-inoculated and mock-treated. The antiviral effects of GUE were evaluated on the basis of diarrhea score, histopathological lesions in the small intestines, quantitation of fecal virus shedding, and expressions of different transcription factors and cytokines. The results obtained from the groups treated with GUE were compared with those of the mock-inoculated and mock-treated groups. Piglets were also observed for signs of adverse effects during drug administration. The studies performed were approved by the University Animal Care Committee (CNU IACUC-YB-R-2009-15).

### Evaluation of fecal consistency

Fecal consistency was examined daily after the inoculation of K85 strain to evaluate the antiviral effect of GUE. A 5-point rating system was used to evaluate fecal consistency: 0, normal; 1, pasty; 2, semi-mucoid; 3, liquid; and 4, profuse diarrhea [37].

### Fecal specimens and extraction of viral dsRNA

Fecal samples were collected daily and were diluted 10 times with 0.01 M phosphate-buffered saline (PBS, pH 7.2). The suspensions were then vortexed for 30 s, centrifuged (1200 x g for 20 min), and then the supernatants were collected and stored at -80°C until used. A starting volume of 200 μl of centrifuged 10% fecal suspensions was used for RNA extraction by an AccuPrep® Viral RNA Extraction Kit (Bioneer, Daejeon, South Korea) according to the manufacturer’s instructions.

### Sample collection

All piglets were immediately necropsied after euthanasia or death. The intestinal tracts were removed from the abdominal cavities and the small and large intestinal contents were collected at necropsy. Each part of small and large intestines was sampled and fixed with 10% buffered formalin solution. Serial 4 μm sections from paraffin embedded blocks were stained with Mayer’s hematoxyline and eosin, and examined microscopically. In a blind fashion, histological evaluation was performed on coded samples, and a comparison was made with the sections from the mock-inoculated and mock-treated, or virus-inoculated but mock-treated controls. Scoring for the small intestinal changes were based on the average villi/crypt (V/C) ratio plus the grade of epithelial cell desquamation, which was measured as follows: V/C ratio, 0 = normal; (V/C ≥6:1), 1 = mild; (V/C = 5.0 – 5.9:1), 2 = moderate; (V/C = 4.0 – 4.9:1), 3 = marked; (V/C = 3.0 – 3.9:1), 4 = severe; (V/C ≤3.0:1), and desquamation grade, 0 = normal (no desquamation), 1 = mild (a few desquamated cells of tip villous epithelium), 2 = moderate (desquamation of upper villous epithelium), 3 = marked (desquamation of lower villous epithelium), 4 = severe (desquamation of crypt epithelium). Ten randomly selected villi and crypts on intestinal histological sections were chosen to determine the mean lesion changes similar to methods described previously [24].

### Real-time RT-PCR using SYBR Green chemistry

Using a primer pair specific to VP6 gene of RVA, a one-step real-time RT-PCR assay based on SYBR Green detection was carried out to quantify the RNA of RVA in the fecal samples, as described previously [24]. Briefly, total RNA extracted from each fecal sample was processed using a Corbett Research Rotor-Gene Real-Time Amplification System (Corbett Research, Mortlake, Australia) and SensiMix one-step RT-PCR with SYBR Green (Quantace, London, UK). The reaction mixture was composed of 5 μl RNA, 12.5 μl SensiMix one-step mixture, 1 μl each of 0.5 M forward and reverse primer, 0.5 μl of 50×SYBR Green solution, 0.5 μl of RNase inhibitor (final concentration: 10 units), 0.5 μl of MgCl_2_ (final concentration: 4.0 mM), and 4 μl of RNase free water. Reverse transcription was performed at 50°C for 30 min, followed by 95°C for 15 min to activate the DNA polymerase and 45 three-step cycles: 95°C for 15 s, 51°C for 30 s, and 72°C for 1 min. Using the Rotagene 6000®, the amount of RVA-specific RNA in the fecal samples was quantified based on the standard curve derived from serial 10-fold dilutions of the in vitro transcription of complementary RNA (cRNA). The highest amplification rate was defined automatically in the initial exponential phase. A direct relationship between the cycle number and the log concentration of RNA molecules initially present in the RT-PCR reaction was evident with regard to the crossing points resulting from amplification curves and this threshold. Rotogene 6000® software was used to set up a standard curve that allowed the determination of concentration of RNA present in the samples using a linear regression analysis of the data.

### Measurement of relative mRNA expression levels of p38, c-Jun N-terminal kinase (JNK), nuclear factor-kappa B (NFkB), interleukin (IL)8, IL10, interferon-beta (IFN-β), IFN-γ and tumor necrosis factor-alpha (TNF-α) in spleen, duodenum, jejunum, and ileum

Sample preparation of spleen and each part of small intestine for extracting total RNA was carried out as described elsewhere [38,39]. Total RNA was extracted using TriReagent® RNA Isolation Reagent (Molecular Research Center, Cincinnati, OH, USA). Isolated RNA was reverse transcribed using QuantiTect® Reverse Transcription Kit (Qiagen, Valencia, CA, USA) according to the manufacturer’s instructions. To reduce discrepancies, all samples were transcribed concurrently in reverse transcriptase efficiency. Real-time PCR primers for porcine p38, JNK, NFκB, IL8, IL10, IFN-β, IFN-γ, TNF-α and β-actin were designed as described elsewhere [38]. mRNA expression levels of p38, JNK, NFκB, IL8, IL10, IFN-β, IFN-γ and TNF-α were quantified by real-time PCR assay using Rota-gene 6000® (Corbett Research, Sydney, NSW, Australia) with 1 μg of cDNA. Twenty-five μL real-time PCR reactions were carried out using SensiMix one-step RT-PCR with SYBR Green (Quantace, London, UK) according to manufactures instructions. The qPCR conditions were performed as described elsewhere [38]. The relative gene expression was assessed by 2-2-ΔΔCt, where ΔCt = Ct of target genes (NF-κB, IL8, IFN-β, TNF-α) - Ct of internal control gene (β-actin). ΔΔCt = ΔCt of samples for target gene- ΔCt of the calibrator for the target gene.

### Statistical analysis

The data gathered from this experiment were written as mean ± standard deviation (SD). To compare between groups, means of the different parameters were analyzed for variance. In order to evaluate the significant differences between the groups, data was analyzed with Tukey’s multiple comparison tests using Minitab Statistical Software 13.20 (Minitab, State College, PA, USA), and the level of significance was selected at *p* < 0.05. Determinations of the One-way ANOVA polynomial orthogonal contrast for the pro-inflammatory markers were performed using IBM® SPSS® Statistics Version 20.0 (Chicago, Illinois, USA).

## Competing interests

The authors declare that they have no competing interests.

## Authors’ contributions

MMA, KHJ, WSL and KOC designed and conceived the experiment, participated in data interpretation and wrote the manuscript. JGP, EHR, JYK, YJJ, DSK, MH, KYS, JHL and SIP contributed to the experimental design and performed experiments. HJK, YBR and SJP extracted and provide the test material. All authors read and approved the final manuscript.
